# Cutaneous manifestations associated with immune checkpoint inhibitors

**DOI:** 10.3389/fimmu.2023.1071983

**Published:** 2023-02-20

**Authors:** Tomoya Watanabe, Yukie Yamaguchi

**Affiliations:** Department of Environmental Immuno-Dermatology, Yokohama City University School of Medicine, Yokohama, Japan

**Keywords:** CTLA-4, cutaneous manifestation, epidemiology, immune checkpoint inhibitors, immune-related adverse events, PD-1, PD-L1

## Abstract

Immune checkpoint inhibitors (ICIs) are monoclonal antibodies that block key mediators of tumor-mediated immune evasion. The frequency of its use has increased rapidly and has extended to numerous cancers. ICIs target immune checkpoint molecules, such as programmed cell death protein 1 (PD-1), PD ligand 1 (PD-L1), and T cell activation, including cytotoxic T-lymphocyte-associated protein-4 (CTLA-4). However, ICI-driven alterations in the immune system can induce various immune-related adverse events (irAEs) that affect multiple organs. Among these, cutaneous irAEs are the most common and often the first to develop. Skin manifestations are characterized by a wide range of phenotypes, including maculopapular rash, psoriasiform eruption, lichen planus-like eruption, pruritus, vitiligo-like depigmentation, bullous diseases, alopecia, and Stevens-Johnson syndrome/toxic epidermal necrolysis. In terms of pathogenesis, the mechanism of cutaneous irAEs remains unclear. Still, several hypotheses have been proposed, including activation of T cells against common antigens in normal tissues and tumor cells, increased release of proinflammatory cytokines associated with immune-related effects in specific tissues/organs, association with specific human leukocyte antigen variants and organ-specific irAEs, and acceleration of concurrent medication-induced drug eruptions. Based on recent literature, this review provides an overview of each ICI-induced skin manifestation and epidemiology and focuses on the mechanisms underlying cutaneous irAEs.

## 1 Introduction

Immune checkpoint inhibitors (ICIs), which are monoclonal antibodies that block key mediators of tumor-mediated immune evasion, were initially approved for treating patients with unresectable malignant melanoma (MM) in 2014, and their frequency of use has rapidly increased in numerous cancers. ICIs target immune checkpoint molecules, such as programmed cell death protein 1 (PD-1), PD ligand 1 (PD-L1), and T cell activation, including cytotoxic T-lymphocyte associated protein-4 (CTLA-4) (1). Anti-PD-1 agents (cemiplimab, dostarlimab nivolumab, pembrolizumab, and tislelizumab), anti-PD-L1 agents (atezolizumab, avelumab, and durvalumab), and anti-CTLA4 agents (ipilimumab and tremelimumab) have been approved by the Food and Drug Administration and European Medicines Agency (**Table 1**). PD-1 is an immune checkpoint receptor expressed on antigen-stimulated T-cells, and PD-L1 is a ligand of PD-1 (2). In contrast, CTLA-4 is an inhibitory receptor expressed on the surface of activated T cells that prevents the binding of CD28 to CD80 and CD86, which are stimulatory receptors. Blockage of PD-1 and/or CTLA-4 can lead to the stimulation and augmentation of anti-tumor effects *via* the activation of tumor-specific cytotoxic T-cells and inhibition of regulatory T cells (Tregs) (2). These treatments constitute one of the most effective strategies for anti-cancer therapy (3).

**Table 1:** Immune checkpoint inhibitors approved by the Food and Drug Administration and European Medicines Agency.

Immune checkpoint inhibitor	Trade name^®^	Target for immunotherapy
Ipilimumab	Yervoy^®^	CTLA-4
Nivolumab	Opdivo^®^	PD-1
Pembrolizumab	Keytruda^®^	PD-1
Cemiplimab	Libtayo^®^	PD-1
Dostarlimab	Jemperli^®^	PD-1
Tislelizumab	No data	PD-1
Atezolizumab	Tecentriq^®^	PD-L1
Avelumab	Bavencio^®^	PD-L1
Durvalumab	Imfinzi^®^	PD-L1

CTLA-4, cytotoxic T-lymphocyte antigen 4.

PD-1, programmed cell death 1.

PD-L1, programmed cell death ligand 1.

However, alterations in the immune system induced by these drugs can lead to various immune-related adverse events (irAEs) specific to ICI treatment. IrAEs can affect multiple organs such as the skin, thyroid gland, adrenal glands, pituitary gland, gut, liver, and lungs (4, 5). Among these, cutaneous irAEs are the most common and often the first to develop (6). To achieve the most favorable outcomes for patients with cancer, an early and accurate diagnosis of irAEs is essential for management, including discontinuation of ICIs and/or the addition of immunosuppressive agents such as systemic corticosteroids. Therefore, dermatologists should be aware of various types of cutaneous irAEs, regardless of whether they are common or rare. In contrast, the mechanism of cutaneous irAEs remains unclear; however, several hypotheses have been proposed based on recent findings.

In this review, we focus on the clinical presentations, mechanisms, and management of various cutaneous irAEs.

## 2 The function of PD-1 and CTLA-4

PD-1 is an inhibitory receptor expressed on the surface of activated T and B cells that induces and maintains peripheral tolerance against self-reactive T cells (7, 8). PD-1 interacts with PD-L1 and PD-L2, which are expressed on antigen-presenting cells (APCs) and tumor cells, resulting in the suppression of T-cell activation and tumor-mediated immune evasion. Inhibition of PD-1 enhances T cell effector function and activation of B cells and natural killer cells, while PD-1 blockade inhibits the suppressive function of Tregs in anti-tumor immunity. Furthermore, PD-L1 and PD-L2 play different roles in the immune response (9). In APCs, stimulation with interferon (IFN)-γ and interleukin (IL)-17A strongly induces PD-L1 expression, whereas PD-L2 expression is induced by stimulation with IL-4. PD-L1 plays an important role in Th1 and Th17 type immunity, while PD-L2 is associated with Th2 type immunity. Therefore, PD-1 blockade may shift the immune balance toward a Th1/Th17 response (9). Furthermore, a recent study revealed that the binding of PD-1, PD-L2, and PD-1–PD-L1 triggered the clustering of PD-1 with T cell receptor (TCR), resulting in the formation of TCR-PD-1-PD-L2 signalosomes. This signalosome suppresses T-cell responses. Similar to the effect of anti-PD-L1 agents, PD-L2 blockade may exert anti-tumor effects, although no therapeutic agents target PD-L2 (10). In the future, anti-PD-L2 agents are expected to be used to treat all types of cancer. CTLA-4 is expressed on the surfaces of activated T cells and Tregs. It can bind to B7 molecules (CD80/86) on APCs with a higher affinity and impede CD28 and B7 binding, suppressing T-cell activation by reducing IL-2 and IL-2 receptor expression (11, 12). Moreover, CTLA-4 expression in Tregs mediates immune inhibitory effects (13). CTLA-4 blocking impedes the binding of CTLA-4 to B7 and induces the binding of CD28 and B7 to reactivate T cells. It also decreases the immune-inhibitory effects of Tregs and further reduces the number of Tregs in tumor tissues *via* antibody-dependent cellular cytotoxicity (ADCC) (14).

Tumors and the tumor microenvironment (TME) express multiple inhibitory pathways and related molecules, resulting in T-cell dysfunction and immune escape. Although the blockage of PD-1 and/or CTLA-4 can promote the activation of T cells and exert an effective anti-tumor function, the exuberant activation of self-reactive T cells with the resultant autoimmunity is presumed to be an irAE (8).

## 3 Mechanisms of cutaneous irAEs

The pathophysiological mechanisms of ICI-induced cutaneous irAEs are mainly unknown; however, skin manifestations are thought to occur *via* several immunological mechanisms. The proposed mechanisms include (1) activation of T cells against common antigens in tumor cells and normal tissues, (2) increased release of inflammatory cytokines and antibodies associated with immune-related effects in specific tissues and organs, (3) association with specific human leukocyte antigen (HLA) variants and organ-specific irAEs, and (4) acceleration of concurrent medication-induced drug eruptions.

### 3.1 Activation of T cells against common antigens in target tumor cells and normal tissues

This mechanism is involved in the cross-reactivity between antigens on ICI-targeting tumor cells and self-antigens in normal tissues. Several studies have shown an association between the appearance of vitiligo, a cutaneous irAE, and the response to treatment with ICIs in patients with melanoma (15–18). Vitiligo is associated with cross-reactivity between melanoma-related antigens and the melanocytes in normal tissues, both of which are possible targets of ICI-induced immune responses (19). In addition, the onset of bullous pemphigoid (BP) may be caused by cross-reactivity between the skin basement membrane and the targeting of BP180 on tumor cells (20). In the analysis of patients with non-small cell lung cancer (NSCLC) treated with anti–PD-1 agents, T cells that recognize both lung tumor tissues and antigens in normal skin simultaneously target both organs. These antigens can stimulate CD4+ and CD8+ T-cells *in vitro*. Furthermore, antigen-specific T cells detected in the peripheral blood were found in the skin lesions and lung tumor tissues of patients treated with an anti-PD-1 agent. Therefore, T-cell clones can interfere with autoimmunity-related skin toxicity in patients with NSCLC treated with anti–PD-1 agent, as well as with tumor regression in patients who respond well to treatment (21).

These reports indicate that the development of cutaneous irAEs is associated with the blockade of common antigens that are co-expressed on both tumor cells and the dermo-epidermal junction and/or other parts of the skin. However, not all tumor tissues have potent neoantigens, and tissue-specific antigens can, in principle, support strong anti-tumor T cell responses with autoimmunity as a toxic skin effect.

### 3.2 Increased release of proinflammatory cytokines and antibodies *via* activation of T cells and B cells

This mechanism may involve various immune cells, such as T and B cells. The blockade of PD-1 and CTLA-4 enhances Th1 and Th17 cell activity (22, 23). Th17 cells produce IL-17A and IL-22, which encourage neutrophil recruitment and the proliferation of epidermal keratinocytes. Thus, ICIs can promote a secondary increase in pro-inflammatory cytokines *via* Th17 cells, resulting in the exacerbation or induction of psoriasis. At the onset of ICI-induced lichen planus (LP), anti-PD-1 agents increase T cell proliferation and IFN-γ and IL-2 production in patients with oral LP (24). It has been shown that nivolumab treatment upregulated granzyme B and IFN-γ in the responding lesions in patients with metastatic melanoma (25). PD-1 is also expressed on major human B-cell subsets, including naive and memory B cells, and the expression of PD-L1 is induced by TLR9 activation. Blockade of the PD-1/PD-L1 pathway increases B-cell activation, proliferation, and production of disease-specific autoantibodies, such as anti-BP180 antibody which is involved in BP (26). These reports suggest that the increased release of proinflammatory cytokines, such as IL-2, IL-17A, and IL-22, and B cell activation with the production of autoantibodies by ICIs treatment are associated with immune-related damage in specific tissues and organs.

### 3.3 The association with HLA variants and the specific irAEs

Specific HLA variants can serve as useful markers of autoimmune diseases. Indeed, the frequency of specific HLA is higher in patients with irAEs than in healthy controls (27, 28). Among cutaneous irAEs, 102 patients with metastatic cancer who received ICIs treatment were significantly associated with HLA-DRB1*11:01 and pruritus (OR = 4.53, X_2_ 1,95 = 9.45, *P* < 0.01) (29). This result indicates that HLA-DRB1*11:01 may be a useful predictive marker for the development of pruritus in patients treated with ICIs, suggesting a genetic etiology for irAEs. However, the specific mechanism underlying HLA-associated irAEs remains unclear. Meanwhile, a large cohort study of 530 patients who received ICIs revealed that irAEs in particular organs and tissues might be associated with certain HLA types (HLA-DRB3*01:01 and thrombocytopenia, HLA-DPB1*04:02 and hypokalemia/hyponatremia, leukopenia and anemia, HLA-A*26:01 and bilirubin elevation); however, HLA heterogeneity has no significant influence on the occurrence of irAEs. In contrast, organ-specific irAEs are strongly involved in multiple HLA variants (27, 28). The molecular mimetic process is tissue-specific, so HLA that presents certain self-peptides may only be associated with some subtypes of irAE. Therefore, HLA types might be useful biomarkers in irAE risk assessments, but it is difficult to identify the association between HLA variants and the occurrence of irAEs. Heterogeneity-inducing mechanisms, such as epitope spread, loss of self-tolerance, and increased inflammatory cytokines, influence the different subtypes of irAEs (30). Further research is required to identify the role of HLA in risk assessment and the occurrence of irAEs.

### 3.4 Allergic mechanisms

MPR, LP/LP-like eruptions, and SJS/TEN/DIHS/DRESS caused by ICIs are partly considered to involve type IV hypersensitivity reactions. ICIs can induce cutaneous reactions through type IV hypersensitivity. In contrast, patients treated with ICIs develop cutaneous irAEs from other concomitant medications, which may resemble the clinical presentation of cutaneous irAEs (31). The administration of ICIs may have triggered an immune response to concomitant medications that were previously tolerated, resulting in the induction of cutaneous irAEs. Indeed, in a retrospective study, 80% of patients who developed lichenoid eruptions after treatment with anti-PD-1/PD-L1 agents simultaneously consumed drugs previously reported to induce lichenoid eruptions (32). The detailed mechanisms remain unclear, but it is speculated to be due to the enhancement of inflammatory response *via* the activation of the immune system, including T cells and APCs, and the inhibition of the suppressive function of Tregs by PD-1 blockage (9, 32). Thus, ICIs may accelerate concurrent medication-induced drug eruptions.

## 4 Epidemiology of cutaneous irAEs

The incidence of cutaneous irAEs ranges from 30 to 60% in patients treated with ICIs (33–37). In contrast, the frequency of cutaneous irAEs differed according to the ICI administered. Anti-CTLA-4 monotherapy has a higher incidence of cutaneous irAEs (44-59%) than anti-PD-1 (34-42%) and anti-PD-L1 monotherapy (up to 20%), whereas combination therapy with anti-PD-1 and anti-CTLA-4 agents has the highest incidence (59−72%) (38, 39). In the severity analysis, cutaneous irAEs were observed in approximately 25% of patients treated with anti-CTLA-4 agents, of which 2.4% were grade 3 and 4 (severe to life-threatening) (40). The incidence of cutaneous irAEs of grades 3 and 4 is much higher during treatment with anti-PD-L1 monotherapy (7.2%) than with anti-PD-1 monotherapy (2.3%) or anti-CTLA-4 monotherapy (4.7%) (41). In combination therapy, anti-PD-L1 and anti-CTLA-4 therapies were associated with the highest incidence (14.5%) compared to anti-PD-1 and anti-CTLA-4 therapies (5.4%) (41). Furthermore, the prevalence of cutaneous irAEs depends on the type of cancer that is treated with ICIs; cutaneous irAEs are more likely to occur in MM than in NSCLC (odds ratio [OR] 1.8, 95% confidence interval [CI] 1.4-2.3) and renal cell carcinoma (RCC) (OR 1.6, 95% CI 1.2-2.1) (42). Different tumor types have different incidences and severities of cutaneous irAEs, even though the same ICIs are used for treatment. The reasons for this observation are not clear, but the TME, immune infiltrate, adaptive immune response, and neoantigen formation may be affected by histology and therefore explain the different skin toxicities (43, 44).

## 5 Severity of cutaneous irAEs

The American Society of Clinical Oncology has established a grading system for the severity of cutaneous irAEs. This system provides appropriate guidelines for the management of cutaneous irAEs according to the involvement of body surface area (BSA) and additional manifestations. Cutaneous irAEs were classified based on histologic and clinical severity and the percentage of BSA involvement (**Table 2**) (45).

**Table 2:** Common Terminology Criteria for Adverse Events.

Grade	Representative Conditions
1	Asymptomatic with macules/papules covering majorly <10% of BSA
2	Macules/papules covering 10% to 30% of BSA can be symptomatic as well as asymptomatic.
3	About >30% of BSA is covered. The appearance of macules/papules with or without symptoms.
4	It is the most severe cutaneous response and can be life-threatening, such as SJS, TEN, and bullous dermatitis involving about >30% of BSA. Intensive care should be taken for proper management.

BSA, body surface area.

SJS, Stevens-Johnson Syndrome.

TEN, toxic epidermal necrolysis.

## 6 Clinical presentation

### 6.1 Maculopapular rash

**Figure 1:**
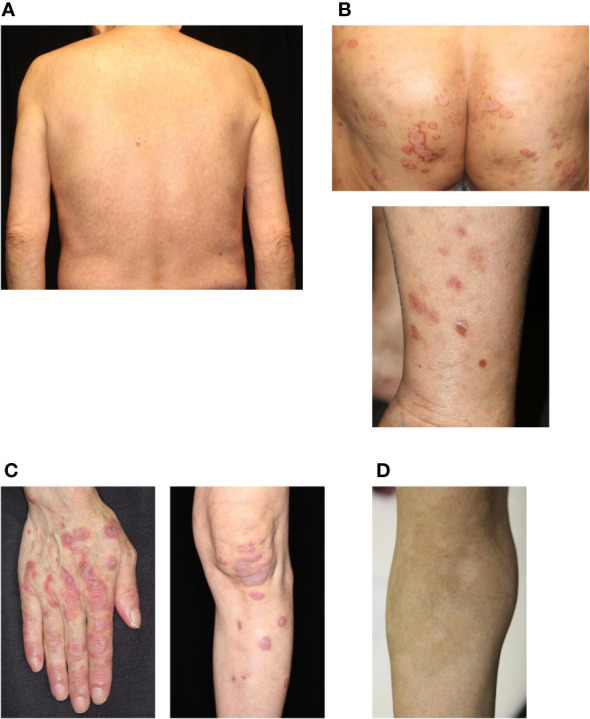
Common cutaneous irAEs. **(A)** Maculopapular rash. Erythematous macules and papules on the trunk. **(B)** Psoriasiform eruption. Scaly keratotic erythema plaques on the gluteal region and lower limbs. **(C)** Lichen planus-like eruption. Erythematous patches with scale and hyperkeratosis on the hands and lower limbs. **(D)** Vitiligo. Depigmented macules developing into plaques on the forearm. irAEs, immune-related cutaneous adverse events. Written informed consent was obtained from the individual(s) for the publication of any identifiable images or data included in this article.

MPR is the most frequent cutaneous irAE and occurs relatively early (39, 46). Rashes reported as irAEs often include MPR. A typical MPR presents as faint erythematous macules and papules that coalesce into plaques. Rashes are generally observed in the trunk and extremities, whereas flexural skin, scalp, palms, and face are rarely involved (**Figure 1A**). Histopathological features revealed superficial perivascular dermatitis with infiltration of CD4-predominant T cells and eosinophils (47, 48). The incidence rate of MPR differed for each of the ICIs. Anti-CTLA-4 agents are associated with a higher risk of MPR than anti-PD-1/anti-PD-L1 agents. Approximately 49–68% of patients receiving anti-CTLA-4 agents can develop MPR of any grade, compared to 20% of patients receiving anti- PD1/PDL-1 agents (40, 48, 49). Skin rashes are widespread; however, almost all patients present with self-limiting MPR, which can be treated with ICIs. However, MPR sometimes occurs as an early manifestation of severe cutaneous irAEs such as the initial presentation of BP, Stevens-Johnson syndrome (SJS)/toxic epidermal necrolysis (TEN), or drug-induced hypersensitivity syndrome (DIHS)/drug reaction with eosinophilia and systemic symptoms (DRESS). The patient should be carefully followed-up for blister formation, mucositis, epidermal detachment, high fever, or swollen lymph nodes from a few days to a week after the onset of such eruptions. In mild cases, MPR is usually treated with topical corticosteroids, emollients, and oral antihistamine drugs. However, systemic corticosteroids (0.5-1 mg/kg/day) are administered in approximately 20% of MPR, and it is estimated that some cases are refractory to treatment with symptomatic therapy (50). Systemic corticosteroids should be considered in patients with systemic symptoms such as fever or widespread erythema multiforme.

### 6.2 Xerosis and eczematous conditions

Xerosis is often observed in patients treated with anti-PD-1 agents. Various types of eczematous dermatitis are often induced by xerosis, particularly during winter. Clinical manifestations often include itchy, poorly demarcated, and erythematous macules and papules that coalesce into plaques and patches on the trunk and extremities, whereas seborrheic dermatitis-like lesions are observed on the face (51). Patients with xerosis are encouraged to moisten their entire body using topical emollients. Eczematous dermatitis is treated with topical corticosteroids, calcineurin inhibitors, and oral antihistamine drugs, depending on the eczematous condition.

### 6.3 Psoriasis/psoriasiform eruption

Psoriasiform dermatitis induced by ICIs can be divided into two types: new-onset psoriasis (*de novo* psoriasis) and worsening existing psoriasis (reactivated psoriasis) (52). Data from the European Network for Cutaneous Adverse Events to Oncologic Drugs revealed that of the 115 ICI-induced psoriasis cases, 30% had reactivated psoriasis, and 70% had *de novo* psoriasis (52). The patients received either anti-PD-1 (86.1%) or anti-PD-L1 (13.9%) agents (53). The typical manifestation appears as scaly erythematous plaques with well-defined borders on the trunk and extremities, while in some cases, palms/soles are involved, and small-sized rashes present as guttate-type psoriasis (**Figure 1B**) (54). Histopathological features resemble spontaneous psoriasis, which shows parakeratosis, loss of the granular layer, acanthosis with elongation of the rete ridges, and perivascular lymphocytic infiltration. However, lichenoid features, spongiosis, and infiltration of eosinophils can be observed in ICI-induced psoriasis (55, 56). ICIs augment Th1 and Th17 activities, resulting in the production of IL-17, which plays an important role in the pathogenesis of psoriasis (22, 23). Interestingly, patients with reactivated psoriasis tend to develop cutaneous findings early after initiation of ICI therapy compared with those with *de novo* psoriasis (50 days *vs*. 91 days) (57). Regardless of the type of psoriasis, ICI-induced psoriasis is treated with topical corticosteroids, vitamin D3 analogs, and narrowband ultraviolet B phototherapy (50, 57). If lesions persist despite these treatments, systemic treatments such as methotrexate, apremilast, retinoids, and biologics can be administered (50, 57–59). However, biologics, particularly tumor necrosis factor-α inhibitors, are contraindicated in patients with active malignancy. In contrast, the use of infliximab for other life-threatening irAEs has been reported, and data on its use in ICI-induced psoriasis are purely descriptive. Hence, IL-17 or IL-23 inhibitors may be preferable.

### 6.4 Lichen planus/lichen planus-like eruption

LP/LP-like eruptions are more often observed in patients treated with anti-PD-1/PD-L1 agents than in those treated with CTLA-4 agents (60, 61). Clinically, LP/LP-like eruptions are observed in 0.5−6% of patients treated with ICIs (48, 62, 63). In a single-institution cohort study, LP/LP-like eruptions affected 17% of patients with metastatic melanoma treated with anti-PD-1 agents (64). Rashes generally show itchy, red to violaceous, flat-topped papules or plaques on the extremities and trunk (**Figure 1C**) (32, 60, 64). Interestingly, unlike spontaneous LP, ICI-induced LP-like eruptions are rarely observed in the oral mucosa (60). Histopathological features included hyperkeratosis, interface changes with dense band-like superficial infiltration of lymphocytes, and keratinocyte apoptosis in the basal layer of the epidermis. Unlike typical LP, epidermal spongiosis, parakeratosis, eosinophils, and necrosis are observed in ICI-induced LP-like eruptions (60, 61, 64). Furthermore, gene expression profiling and immunohistochemical staining showed upregulation of toll-like receptor (TLR) 2 and TLR4 and increased CD14+ and CD16+ monocytes in patients with lichenoid dermatitis. Thus, the innate immune response may be involved in the onset of LP/LP-like eruptions *via* the activation of CD14/TLR signaling (65). The treatment of LP/LP-like eruptions mainly consists of topical corticosteroids, which allow the continuation of ICIs therapy. In intractable cases, systemic treatment, including oral corticosteroids, retinoids, cyclosporine, and narrowband ultraviolet B phototherapy, has also been reported to be effective in intractable cases (38, 66).

### 6.5 Vitiligo

Vitiligo is an autoimmune disease characterized by loss of melanocyte function in the epidermis. ICI-induced vitiligo is frequently observed in melanoma patients, whereas other cancers are less commonly reported. Vitiligo affects 2–9% of patients with melanoma treated with anti-CTLA-4 agents and 7–11% of those treated with anti-PD-1 agents or combination therapy (38). Unlike idiopathic vitiligo, the clinical features of ICI-induced vitiligo are characterized by its occurrence in photo-exposed areas, which consist of flecked macules that coalesce into patches without koebnerization (**Figure 1D**) (67). In the treatment with anti-PD-1 agents, depigmentation is induced by the activation of CD8+ cytotoxic T cells against melanoma-associated antigens (MART-1/MelanA, gp100, and tyrosinase-related proteins 1 and 2) shared by normal melanocytes and melanomas (18, 68). Unfortunately, no definitive treatment for ICI-induced vitiligo has been reported, and most cases do not improve after ICI discontinuation. However, the onset of depigmentation during ICI therapy is significantly associated with favorable results in melanoma (69, 70).

### 6.6 Bullous diseases

**Figure 2:**
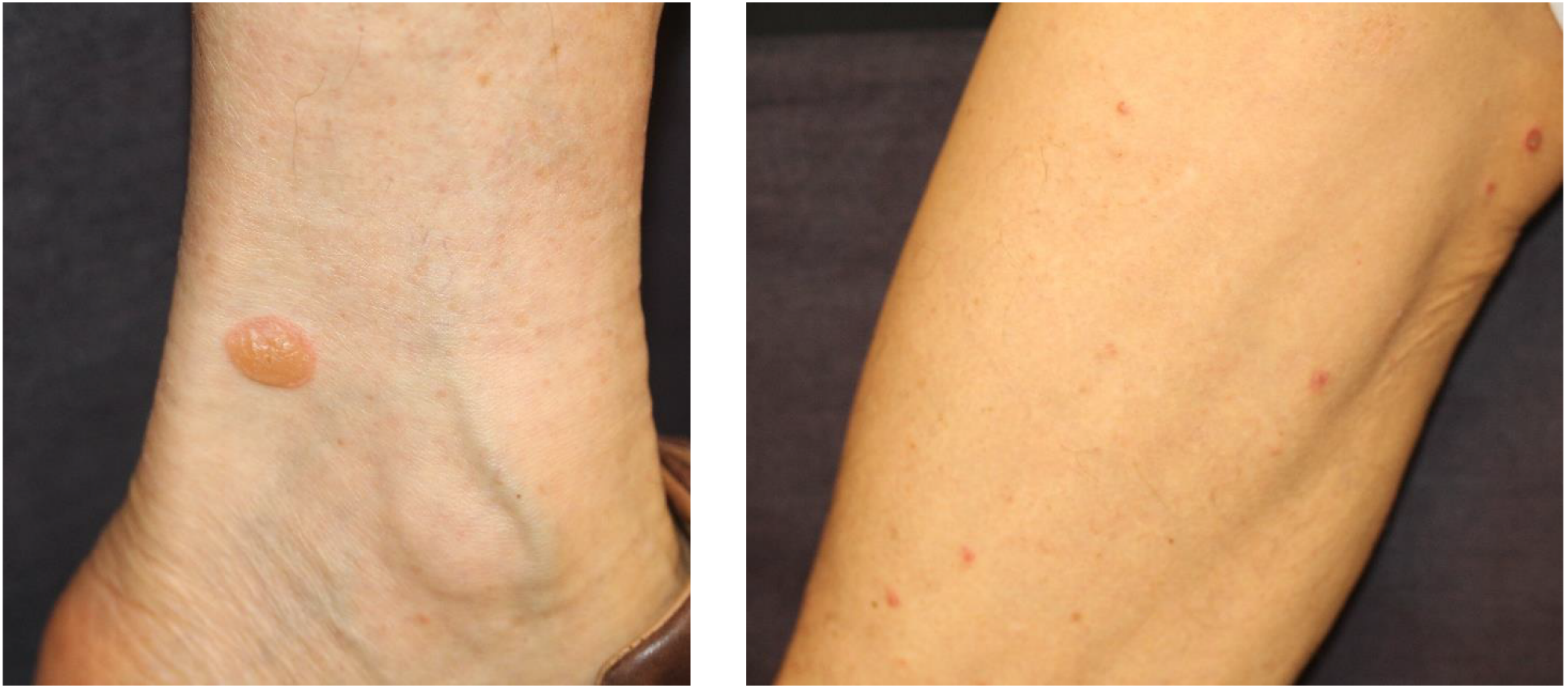
Bullous pemphigoid eruption. Multiple small tense bullae on extremities. Written informed consent was obtained from the individual(s) for the publication of any identifiable images or data included in this article.

Autoimmune bullous diseases are rare cutaneous irAEs characterized by autoantibodies against basement membrane proteins BP180 and BP230 (71–76). The incidence of autoimmune bullous diseases, including BP, bullous lichenoid dermatitis, and linear IgA bullous dermatosis, is approximately 1% in patients administered anti-PD-1/PD-L1 agents (77). The clinical manifestations of ICI-induced BP include tense bullae and erythematous erosions on the trunk and extremities, while involvement of the mucous membrane is less frequent (**Figure 2**) (77, 78). The histopathological features of ICI-induced BP are similar to those of spontaneous BP, which shows subepidermal blistering with eosinophilic infiltration and linear deposition of complement component 3 (C3) and immunoglobulin G (IgG) in the basement membrane zone on direct immunofluorescence (73). Blockade of the PD-1/PD-L1 pathway enhances B cell activation, resulting in the production of autoantibodies such as anti-BP180 antibody and inflammatory cytokines. Moreover, basement membrane components are also expressed in cancers and in the production of autoantibodies against different epitopes (cross-reactivity), thus causing ICIs to induce the development of BP (20). Mild cases are generally treated with a combination of high-potency topical corticosteroids and doxycycline with or without niacinamide (79). Severe cases typically require systemic corticosteroids (0.5−1.0 mg/kg/day prednisone) that are slowly tapered over the course of 1–2 months and the addition of rituximab, which is a B cell-depleting anti-CD20 antibody (77, 79, 80). The development of BP is linked to favorable tumor response to anti-PD-1 agents and decreased mortality (81); however, other studies did not support this finding (20).

### 6.7 Alopecia

**Figure 3:**
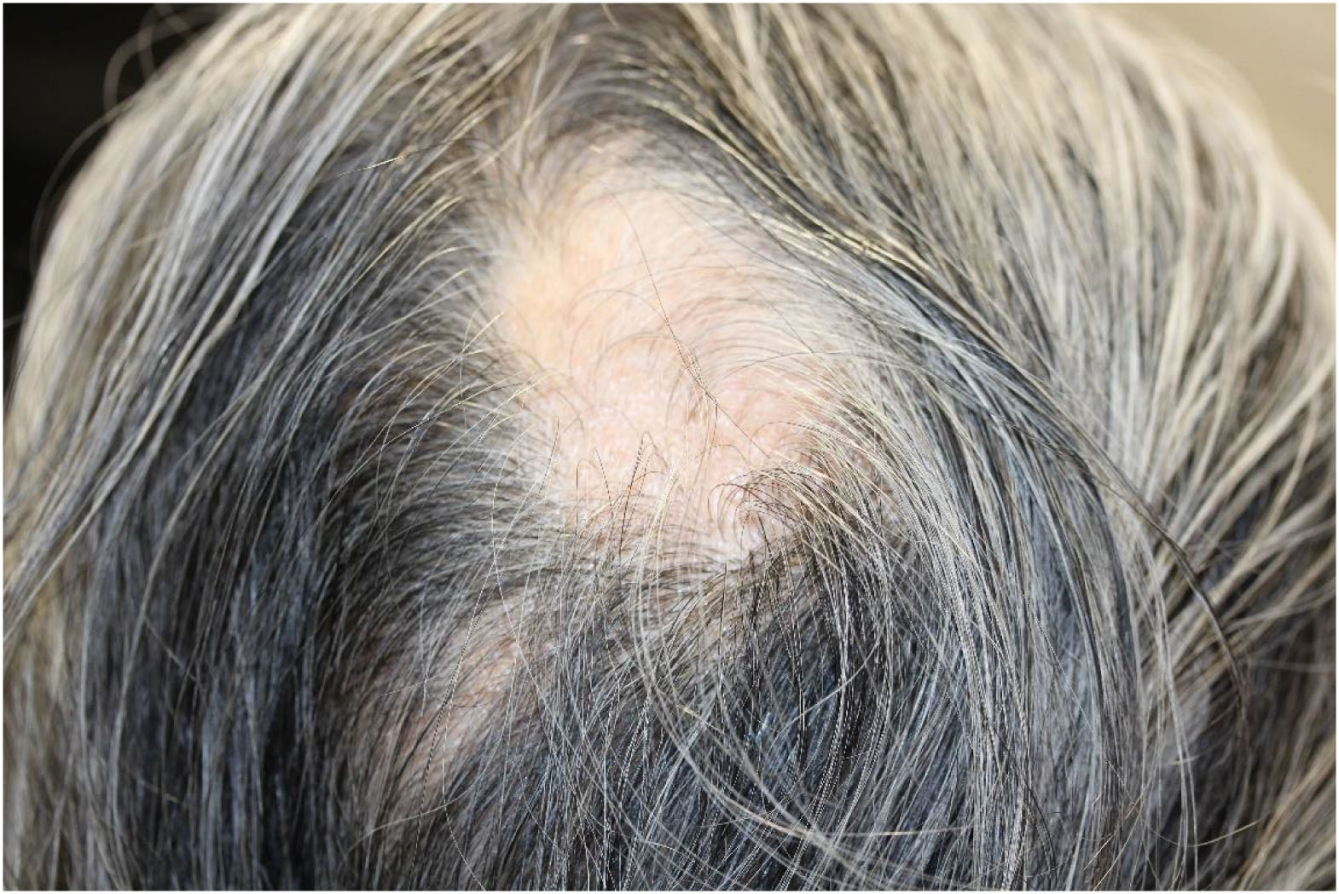
Alopecia areata. Circumscribed patches of hair loss on the parietal region. Written informed consent was obtained from the individual(s) for the publication of any identifiable images or data included in this article.

Alopecia is less frequent but represents a significant proportion of irAEs. The incidence of ICI-induced alopecia is approximately 1–2% (82–84). ICI-induced alopecia shows a phenotype similar to alopecia areata, in which hair follicles are impaired by CD8+ T-cells (85). The clinical manifestations of alopecia vary, ranging from well-circumscribed patches or diffuse hair loss on the scalp to alopecia universalis (**Figure 3**) (86). Histopathological examination revealed a perifollicular lymphocytic inflammation. The hair follicle dermal sheath expresses PD-L1, and PD-1 blockade directly induces alopecia areata, alopecia totalis, or alopecia universalis *via* CD4+ and CD8+ T cell–mediated immune response (85). Alopecia is usually treated with intralesional and topical corticosteroids as well as systemic immunomodulators (86, 87).

### 6.8 Pruritus

Pruritus is the second most common cutaneous irAE associated with ICIs. The overall incidence of pruritus is reported to be 13–20% (38, 88). Symptoms are commonly grade 1 or 2 in severity, with high-grade pruritus rarely reported (<2% of patients) (46, 88). In another report, pruritus affected 14−47% of patients, of whom the highest incidence was observed in patients receiving anti-CTLA-4 agents (25−36%) and combination therapy (33−47%) (38). The onset of pruritus is associated with a specific HLA. In an analysis of 102 patients receiving anti-PD-1, anti-CTLA-4, or combination therapy, a significant correlation was found between HLA-DRB1*11:01 and pruritus (29). Pruritus often complicates other skin changes such as erosions, ulcerations, hyperpigmentation, or prurigo nodules, but it can also occur independently of any other skin changes. Pruritus is commonly observed on the trunk and scalp, whereas the face, soles, anterior neck, and genitalia are rarely involved (38, 89). Pruritus is mainly treated with oral antihistamine drugs and emollients with or without topical corticosteroids (35, 90). In grades 3 and 4 severity, severe itchiness affects the quality of life and sometimes requires discontinuation of ICIs (35, 90).

### 6.9 Scleroderma

**Figure 4:**
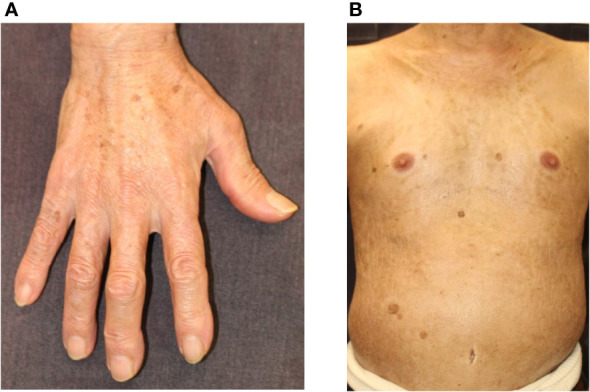
Scleroderma. **(A)** Skin thickening and hardening causing the stiffness of the fingers and hands. **(B)** Skin thickening and hardening with pigmentation on the trunk. Written informed consent was obtained from the individual(s) for the publication of any identifiable images or data included in this article.

ICI-induced scleroderma-like lesions are rare cutaneous irAEs (**Figure 4**). Terrier et al. summarized 10 cases (5 males and 5 females), which consisted of 6 cases of systemic sclerosis (2 limited and 4 diffuse types) and 4 cases of morphea (2 localized and 2 generalized types) (91). The ICIs administered to these 10 patients included nivolumab in 4 cases and pembrolizumab in 6 cases (91). Interestingly, sclerotic skin changes were observed more rapidly with pembrolizumab than with nivolumab. This result indicates that inhibition of PD-1/PD-L1 may be associated with the onset of scleroderma-like lesions. Indeed, blockade of the PD-1/PD-L1 pathway induces macrophage polarization to the profibrotic M2 type, resulting in excessive production of extracellular matrix proteins *via* fibroblast activation (92). Th17 is also involved in the pathogenesis of systemic sclerosis, and blockage of PD-1 leads to a shift in the immune balance toward a Th1/Th17 response (9). Regarding the treatment of scleroderma-like lesions, six patients with generalized skin lesions were treated with high-dose corticosteroids, and almost all showed improvement in skin thickness (92).

### 6.10 SJS/TEN/DIHS/DRESS

**Figure 5:**
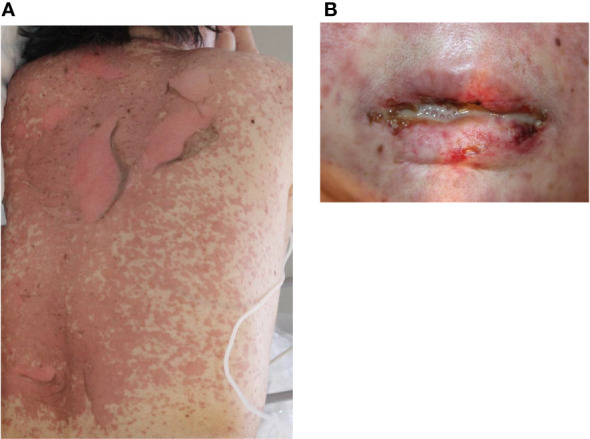
Toxic epidermal necrolysis. **(A)** Erythematous macules, bullae, and erosions on the trunk. **(B)** Hemorrhagic erosion of the lip. Written informed consent was obtained from the individual(s) for the publication of any identifiable images or data included in this article.

Severe cutaneous drug eruptions, including Stevens-Johnson syndrome (SJS) and toxic epidermal necrolysis (TEN), are rare with ICIs treatment, but life-threatening cutaneous adverse drug reactions are characterized by high fever, widespread detachment of the epidermis, and erosions, and mucositis. However, the incidence of ICI-induced SJS/TEN remains unknown. To date, 20 cases of SJS/TEN have been reported, including 12, six, and two cases associated with the use of pembrolizumab, nivolumab, and atezolizumab, respectively (37). The clinical and histopathological features of ICI-induced SJS/TEN were similar to those of SJS/TEN caused by other drugs (**Figure 5**). It has been speculated that PD-L1 is typically undetectable in epidermal keratinocytes, but ICI treatment increases PD-L1 expression, which induces apoptosis of PDL-1 expressing keratinocytes by activated cytotoxic CD8+ T cells (93). In addition, ICI-induced skin damage shows a similar gene expression profile as SJS/TEN from other causative drugs, with increased expression of inflammatory chemokines, cytotoxic mediators (perforin and granzyme B), and apoptosis-promoting molecules (Fas Ligand) (94, 95). Furthermore, the dysfunction of Tregs and enhancement of co-stimulatory factors are associated with SJS/TEN pathogenesis. Degranulation of CTL and NK cells, which induces apoptosis of keratinocytes, has been implicated in the association between CD49/NKG2C and HLA-E. Additional implicated factors include Fas/FasL, PD-L1-expressing T cells and epidermal cells, and CD40/CD40L interactions at the dermal-epidermal junction (96, 97). In the management of SJS/TEN, it should be discontinued. Treatment with high-dose corticosteroids (0.5-1.0 mg/kg/day prednisone) is recommended because the mechanism of adverse events involves T-cell immunodirected toxicity. In addition to systemic corticosteroids, adjuvant therapies based on a combination of immunosuppressive agents, such as oral cyclosporine, intravenous immunoglobulin (IVIG), and/or plasmapheresis therapy, have been proposed (98). However, the effect of immunosuppressive agents on cancer is unknown; therefore, the decision to use immunosuppressive agents should be made in consultation with the oncologist in charge. The mortality rates of SJS and TEN are 10% and 50%, respectively (94).

Drug-induced hypersensitivity syndrome (DIHS)/drug reaction with eosinophilia and systemic symptoms (DRESS) can also be induced by ICIs but are rare cutaneous irAEs. In the three published cases of ICI-induced DIHS/DRESS, the causative ICIs consisted of one case of use of nivolumab and one case of use of ipilimumab (99–101). Patients developed renal and hepatic involvement, although steroid-responsive multiorgan dysfunction could have occurred due to either DIHS/DRESS or direct ICIs toxicity. For the management of DIHS/DRESS, discontinuation of ICIs and administration of systemic corticosteroids (1.0 mg/kg/day prednisone) a required. In contrast to SJS/TEN, systemic corticosteroids should be tapered slowly over at least 6–8 weeks to reduce the risk of recurrence.

## 7 Time to onset of cutaneous irAEs

**Figure 6:**
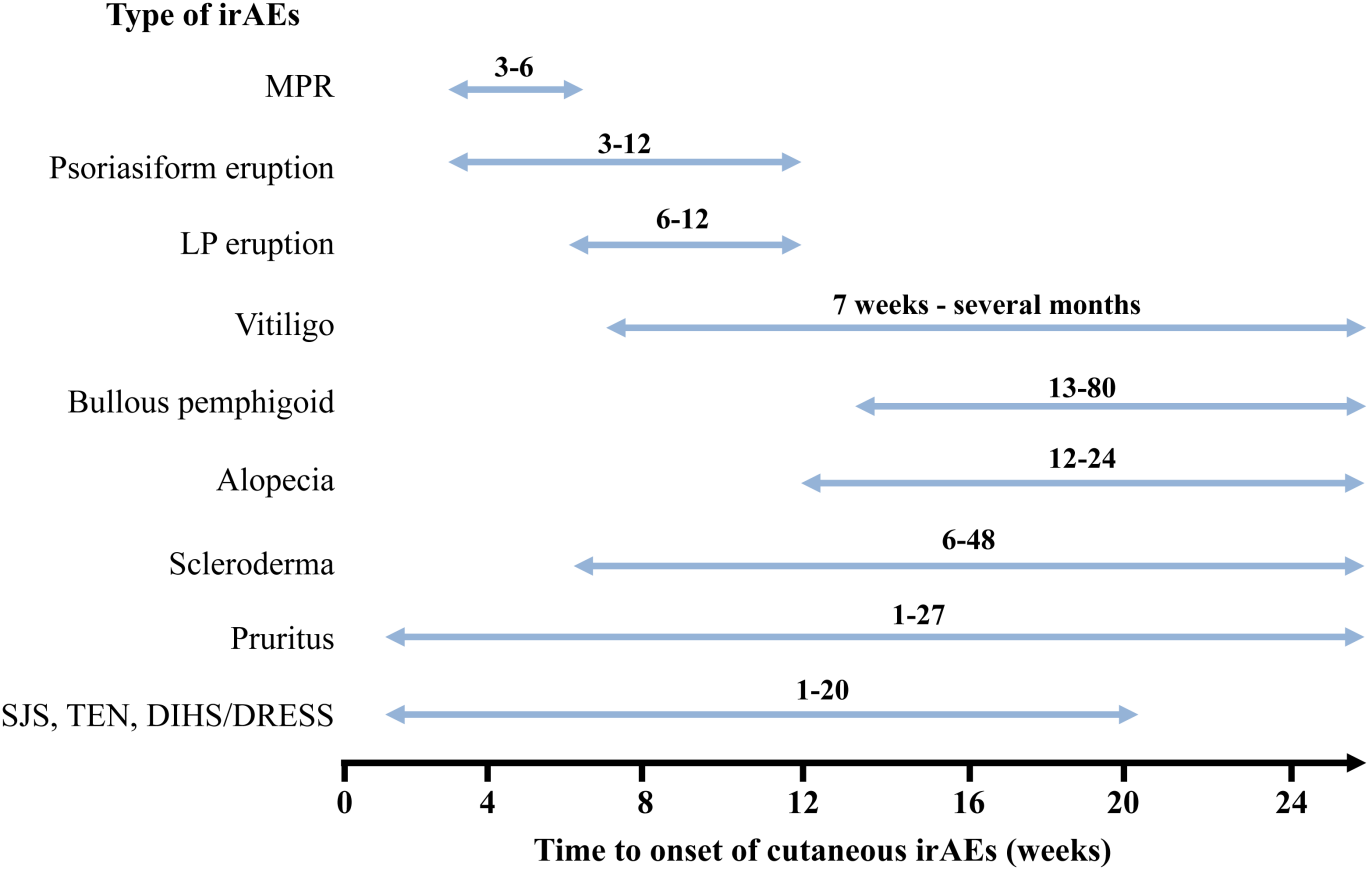
Time to onset of cutaneous immune-related cutaneous adverse events. MPR, Maculopapular rash; SJS, Stevens-Johnson Syndrome; TEN, toxic epidermal necrolysis; DIHS, Drug-induced hypersensitivity syndrome; DRESS, Drug reaction with eosinophilia and systemic symptoms; irAEs, immune-related cutaneous adverse events.

The time from the initiation of ICIs treatment to the onset of irAEs is generally weeks to months. Among the various irAEs, cutaneous irAEs are the earliest complications to develop (47, 102). However, the duration from the initiation of ICIs to the onset of cutaneous irAEs differs depending on the type of skin rash. **Figure 6** summarizes the time to the onset of each cutaneous irAE. MPR occurs within the first 3–6 weeks of ICIs treatment initiation (40, 48, 49). Similar to MPR, psoriasiform rash appears three weeks after the initiation of ICIs treatment (54, 57). In contrast, other studies have shown that the time from ICIs treatment initiation to psoriasis development ranges from 5− to 12 weeks (53, 103). These differences may be due to *de novo* or reactivated psoriasis (57). The onset of LP/LP-like eruptions occurs later than that of psoriasiform rash and MPR, with an onset ranging from 6 to 12 weeks (34, 48). In another report, LP/LP-like eruptions occurred anywhere from 3 to 52 weeks after the initiation of ICIs treatment (32). The occurrence of vitiligo is characterized by late onset, with the time to development varying from seven weeks to several months (median onset time of approximately 26 weeks) after ICIs initiation (46, 104, 105). The onset of alopecia is also delayed, occurring within 12–24 weeks of the initiation of ICIs treatment (83). The time to the onset of BP eruptions also varied according to previous reports. Previously, BP eruptions had been reported to occur at 13-15 weeks (48), but more recent studies have reported that they can occur anywhere between 3− and 80 weeks after ICI therapy initiation (71, 72, 78). Pruritus can develop 1− to 27 weeks after initiating ICIs (106). Since the onset of ICI-induced scleroderma is very rare, the detailed time from ICI initiation to onset is unknown. However, in 10 published cases, the time from ICIs therapy initiation to scleroderma onset ranged from 6− to 48 weeks (91, 107–110). The onset of severe cutaneous adverse reactions, including SJS, TEN, and DIHS/DRESS, varies from one − to 20 weeks (94). The average onset time is reported to be 8.9 weeks for SJS and 5.4 weeks for TEN; however, they can occur within a week of the first administration of ICIs (111). Interestingly, different types of skin eruptions can develop in the same individual at different times, depending on the type of lesion.

## 8 Association with cutaneous irAEs and prognosis

Patients who develop irAEs are generally considered to have a high anti-tumor response. In patients with malignant melanoma treated with nivolumab, overall survival was improved in patients who developed irAEs and in those with a higher number of irAEs (16). Interestingly, in a large cohort analysis of the association between cutaneous irAEs and survival rates within six months of anti-PD-1/PD-L1 therapy, the incidence of cutaneous irAEs correlated with decreased mortality (112). Furthermore, cutaneous irAEs often precede irAEs in other organs and are expected to be biomarkers for the development of secondary irAEs and overall survival. Thompson et al. examined the clinical types of cutaneous irAEs and other organ irAEs and found an association between mucosal lesions and overall irAEs as well as psoriasis-like skin rash and endocrine organ irAEs (113). In a retrospective study, lichenoid and spongiotic dermatitis were identified as biomarkers of favorable tumor response in patients receiving anti-PD- 1/PD-L1 therapy (114). Another study focused on the histopathology of cutaneous irAEs and their prognosis. Hirotsu et al. showed associations between vacuolar lesions and pneumonia, psoriasis-like histology and musculoskeletal and multiple other organ irAEs, and bullous histology and ipilimumab–nivolumab combination therapy (114). Spongiform changes and lichenoid reactions are associated with progression-free survival and decreased mortality, whereas vacuolar degeneration and superficial perivascular dermatitis increase the risk of mortality (115).

## 9 Conclusions

ICIs have been approved for many advanced malignancies and will be developed and used for more malignancies in the future. Although cutaneous irAEs are the most common adverse events, most cases are mild (grades 1 and 2), allowing continuous treatment with ICIs. In contrast, rare and severe cutaneous irAEs such as SJS/TEN and DIHS/DRESS should be carefully considered for discontinuation of ICIs and treatment. To achieve the most favorable outcomes for patients with cancer, early and accurate diagnosis of irAEs is essential to implement management steps, including discontinuation of ICIs and/or addition of immunosuppressive agents such as systemic corticosteroids. Furthermore, as recent studies have shown, a detailed diagnosis of cutaneous irAEs may provide useful information regarding patient prognosis and biomarkers for predicting subsequent irAEs and their risk factors. Therefore, dermatologists should be aware of many types of cutaneous features, whether they are common or rare, treatment strategies for cutaneous irAEs, and their mechanisms of action.

## Author contributions

YY contributed to determining the content of each section and edited the manuscript. TW collected data and wrote each section of the manuscript. All authors contributed to the article and approved the submitted version.
